# Effects of a health education intervention on knowledge and attitudes towards chronic non‐communicable diseases among undergraduate students in Jordan

**DOI:** 10.1002/nop2.634

**Published:** 2020-09-29

**Authors:** Maysa H. Almomani, Mohammad Rababa, Fatmeh Alzoubi, Karimeh Alnuaimi, Ahlam Alnatour, Reem A Ali

**Affiliations:** ^1^ Department of Adult Health Nursing Faculty of Nursing Jordan University of Science and Technology Irbid Jordan; ^2^ Department of Community and Mental Health Nursing Faculty of Nursing Jordan University of Science and Technology Irbid Jordan; ^3^ Department of Midwifery Faculty of Nursing Jordan University of Science and Technology Irbid Jordan; ^4^ Department of Maternal and Child Health Nursing Faculty of Nursing Jordan University of Science and Technology Irbid Jordan

**Keywords:** attitudes, chronic non‐communicable diseases, education, health promotion, knowledge, undergraduate students

## Abstract

**Aim:**

To assess the effect of a health promotion course on knowledge and attitudes towards chronic non‐communicable diseases (CNCDs) among undergraduate students in a Jordanian university.

**Design:**

A one‐group pre‐test–post‐test study design was used.

**Methods:**

A convenience sample of 178 undergraduate students registered in a 3‐credit health promotion course was enrolled in the study and completed both pre‐ and post‐tests. This course is offered as an elective course for undergraduate students by the Faculty of Nursing at a large public university in Jordan. Twelve, one‐hour interactive sessions regarding CNCDs topics were delivered over 3 weeks as part of the health promotion course. A computer‐based questionnaire was used to evaluate students’ knowledge and attitudes towards CNCDs including diabetes, hypertension and cancer, before and after undertaking the course.

**Results:**

There was a significant improvement in the overall knowledge (Cohen's *d* effect size (*d*) = 1.08) and attitudes (*d* = 0.62) among all the students, regardless of gender. The overall knowledge and attitudes scores were significantly higher among females in the pre‐test, but the differences in their overall scores became insignificant in the post‐test. Education on specific topics was effective in improving university students’ knowledge and attitudes about health‐promoting behaviours, thereby preventing CNCDs. It is important to incorporate health promotion education regarding CNCDs into university curricula using innovative approaches to enhance healthy behaviours in young adults.

## 1 INTRODUCTION

Chronic non‐communicable diseases (CNCDs) are the leading cause of morbidity and mortality globally. In 2016, CNCDs, including cardiovascular diseases (CVDs), cancers, respiratory diseases and diabetes, accounted for 71 per cent of total deaths worldwide (World Health Organization [WHO], 2018a). In Jordan, there were 36,000 total deaths in 2016 and an estimated 78 per cent of them were from CNCDs, mainly by CVDs (37%), cancer (12%) and diabetes (6%) (WHO, 2018b). The leading cause of morbidity among Jordanians aged 25 years and above include hypertension (28.6%), high cholesterol (39.5%), high triglyceride (56.5%) and diabetes (22.3%) (The Higher Health Council, 2015). These CNCDs share common, causal, behavioural risk factors including physical inactivity, unhealthy diet, smoking and alcohol (WHO, 2018b). Therefore, CNCDs and their associated complications might be preventable making these high morbidity and mortality rates unacceptable.

Primary prevention of CNCDs can be achieved by health promotion interventions that can empower people to enhance control over their own health and environments (Joh et al., 2017; Nayak et al., 2016). Health promotion education can enhance individuals’ lifestyle behaviours and their knowledge and attitudes towards the adoption of healthy behaviours particularly when innovative approaches are used (Abdo et al., 2019; Belogianni & Baldwin, 2019; Nayak et al., 2016; Selvam et al., 2017). University students are vulnerable to engage in health‐compromising behaviours (Almutairi et al., 2018; Joh et al., 2017), thus a distinct priority population for health promotion to enhance responsibility for their own health and minimize the risk of developing diseases later in life. Universities are key locations for cost‐effective and ideal health education on CNCDs to enhance young adults lifelong behaviours, such as exercise and a healthy diet (Abdo et al., 2019; Joh et al., 2017; Nayak et al., 2016; Wang et al., 2013).

Improvements in knowledge and attitudes are significant precursors of behaviour change (Belogianni & Baldwin, 2019). Research indicates that students have poor knowledge and negative attitudes regarding risk factors, preventive measures and complications of CNCDs (Abukhelaif & Alghamdi, 2017; Alsaraireh & Darawad, 2018; Kan’an, 2018; Shah et al., 2016). In Jordan, lack of awareness in terms of health promotion behaviours is a clear problem among university students (Al‐Shara, 2019; Ashraah et al., 2013; Shaheen et al., 2015). For instance, female students had relatively poor knowledge, negative attitudes and poor practice regarding breast cancer (BC) (Alsaraireh & Darawad, 2018, 2019).

Evidence suggests that health education about CNCDs is effective in improving university students’ knowledge, attitudes and healthy lifestyle behaviours (Abdo et al., 2019; Belogianni & Baldwin, 2019; Nayak et al., 2016; Selvam et al., 2017). In this regard, using an interactive approach can help students better engage in learning healthy lifestyles (Ali et al., 2018; Alsaraireh & Darawad, 2019; Joh et al., 2017). The effectiveness of health education was evaluated by two Jordanian studies, revealing a positive effect on improving healthy nutritional habits (Abu‐Moghli et al., 2010) and on improving knowledge, attitudes and practices on BC and breast self‐examination among university students (Alsaraireh & Darawad, 2019). Yet, studies examining the effectiveness of using interactive health education on knowledge and attitudes towards health promotion and prevention of CNCDS among university students in Jordan and surrounding countries are lacking.

In summer 2016, the United Nations Population Fund (UNFPA) had a strategic goal to improve the health of young Jordanians. They collaborated with a large public university in Jordan, which regularly administers a health promotion course (HPC) to undergraduate students, to integrate CNCDs topics within its curriculum using interactive pedagogical approaches. Therefore, the purpose of this study was to assess the effect of the HPC, after integrating CNCDs topics, using a new methodology on improving the knowledge and attitudes about CNCDs among university students in Jordan. In addition to getting the baseline data, the findings from this study may encourage stakeholders in Jordan to integrate education regarding the prevention of CNCDs and adopt such interactive teaching strategies in other courses to improve the knowledge and attitudes of all young adults in Jordan.

## 2 METHOD

### 2.1 Design, setting and sample

A one‐group pre‐test–post‐test study was conducted in a large public university in Jordan that enrols students from all around the country. A convenience multi‐disciplinary sample of undergraduate students who were enrolled in the three‐credit hours HPC during the summer of 2016 was used. This elective course is offered by the university to undergraduate students from different years and all majors, except medicine and nursing majors. Otherwise, the sample included students from all other health science majors available at the university, such as pharmacy, dentistry and applied medical sciences. All students who were enrolled in the HPC and able to read and write Arabic were eligible to be included in the study. Participation was voluntary and those who agreed to participate completed a pre‐test prior to the first session of the course and a post‐test after the completion of the course. The course was offered in Arabic in a classroom setting.

### 2.2 Measurements

A computer‐based questionnaire was used to evaluate students’ health promotion knowledge and attitudes about three major CNCDs, including diabetes mellitus (DM), hypertension and cancer before and after the HPC. The researchers developed the questionnaire after reviewing relevant literature (Abukhelaif & Alghamdi, 2017; Alsaraireh & Darawad, 2018; Lorga et al., 2013; Parmar et al., 2014) and obtaining special training from the UNFPA on designing HPC material on CNCDs. The items in the questionnaire were based on the HPC content on the prevention and control of DM, hypertension and cancer.

The questionnaire consisted of three sections: demographics (8 questions), knowledge (18 questions) and attitudes (18 questions) about CNCDs. Each of the knowledge and attitudes scales consisted of three 6‐item subscales on DM, hypertension and cancer. For knowledge items, a score of 1 was assigned to the correct response, while 0 was assigned to the incorrect responses, which were the “false” and “I do not know” responses, with a total knowledge score of 18. For attitudes items, responses were scored as follows (0 = disagree, 1 = not sure and 2 = agree), yielding a maximum score of 36. Higher scores indicate better knowledge and attitudes.

The questionnaire was developed in Arabic and reviewed by a panel of six assistant and associate professors in nursing who are experts in the topic to establish its content and face validity. The item content validity index (I‐CVI) ranged between 0.83 and 1 and the scales content validity index (S‐CVI) was >0.97 for both knowledge and attitude scales. The questionnaire was piloted for clarity, readability and feasibility on 20 students whose data were not included in the final analysis. The questionnaire was clear and easy to understand, requiring 10–15 min for completion. Internal consistency was also achieved for both knowledge and attitude scales (Cronbach's alpha: 0.78 and 0.85, respectively).

### 2.3 Intervention: the HPC

The HPC is a three‐credit hour elective course offered by the Faculty of Nursing at a public university in Jordan for undergraduate students from all majors, except medicine and nursing majors. The medium of instruction for this course was Arabic. Content related to the CNCDs was integrated into the HPC to cover the definitions, modifiable and non‐modifiable risk factors, signs and symptoms, complications, screening and diagnoses, treatments, preventive measures of the three CNCDs and the related healthy lifestyle modifications. Content related to the CNCDs was delivered in twelve, one‐hour sessions over 3 weeks, by nursing professors who were well‐trained on the content and teaching methodology. Interactive teaching methods that incorporate meaningful interactions among learners themselves and between learners and educators were used to facilitate better knowledge acquisition while facilitating a healthy learning environment (Buehl, 2017). The interactive teaching methods used are given in Table 1.

**TABLE 1:** Pedagogical approaches used

Orientation	The students received an orientation about the interactive teaching methods and all salient information related to the study including significance, purpose, design, procedure and pre and post‐test questionnaires.				
Topics	Icebreaker	Concept mapping	Think, pair and share	Buzz session	Incident process (real/video)
HTN	Ask students to: Think back over their life and identify a moment when dealing with a person with CNCD Share with each other: Challenges when dealing with that person. Behaviours or appearance that could be related to CNDCs. Similarities in their experiences. Form groups (5 students each), based on similarities in answers.	Ask students to: Collect in groups (5–6 students) Think of CNCDs risk factors Draw and discuss a concept map linking risk factors to CNCDs, within their own groups. Draw concept map on the whiteboard (by group leaders). Class discussion of concept maps.	Provide students with a problem‐based question regarding CNCDs Ask each student to: Think about answer. Pair up with a partner Share the solution with the partner. Expand the share into a whole‐class discussion.	Ask students to: Collect in groups (4–5), Discuss a given issue or topic of CNCDs. Appoint one student of each group to write down a group's ideas. Ask each buzz groups to present their report to entire class. Class discussion and feedback.	Provide students with a real case study/or video about CNCDs. Students think of a solution/or plan to control this CNCD. Divide students into small groups (5–6 students) Students share and discuss their solutions within their group. Each group share their solution with other groups, Class discussion and feedback.
DM					
Cancer Healthy lifestyles					

Abbreviations: CNCD/s, chronic non‐communicable disease/s; DM, diabetes mellitus; HP, health promotion;HTN, hypertension.

### 2.4 Procedure

Following approval from the Institutional Review Board and before the course starts, the researchers sent an announcement via email to the students enrolled in the HPC requesting voluntary participation in the study. The announcement clarified the study's purpose and nature, the confidentiality and anonymity of responses, participants’ right to decline participation or withdraw at any time without any penalty and that participants’ pre‐ and post‐tests scores would not be counted towards their final course grades. A written informed consent was obtained from participants during the orientation day. Three days before starting the course, the pre‐test questionnaire was made available to the eligible participants on the e‐learning platform used by the university. At the end of the course, the participants completed the post‐test using the same questionnaire and e‐learning platform. All data were electronically extracted after deleting students’ identifiers and stored in a password‐protected computer, accessible only to the principal investigator.

### 2.5 Data analysis

Data were analysed using SPSS 23. Descriptive statistics were used to describe students’ levels of knowledge and attitudes on CNCDs for the pre‐test, post‐test and demographic data. The paired sample *t* test was used to analyse the differences in students’ levels of knowledge and attitudes about CNCDs before and after the HPC. An independent sample *t* test was used to analyse gender differences in the level of knowledge and attitudes before and after the HPC. The significance level was set at 0.05 and the Cohen's *d* effect size close to *d* = 0.5 was classified as medium and *d* ≥ 0.8 as large.

### 2.6 Ethics

This study was conducted after obtaining the approval of the Institutional Review Board (IRB # 184‐2016) at a large public university in Jordan. A written informed consent was obtained from all participants.

## 3 RESULTS

Out of the 252 students invited to participate in the study, 181 agreed to participate in the study and entered the pre‐test, while 178 completed both pre‐ and post‐tests (response rate = 70.6%, completion rate = 0.98). All students completed the HPC (12 sessions). Students’ mean age was 19.9 years [standard deviation (*SD*) = 1.35 years, range = 18–25 years] and 69.1 per cent (*N* = 123) of them were females. Most students were unemployed (91.6%, *N* = 163), free from chronic diseases (94.9%, *N* = 169) and from northern Jordan (74.16%, *N* = 132). Forty‐six students (25.84%) were from middle and southern Jordan.

The overall knowledge and attitudes scores at the baseline did not differ significantly by socio‐demographic factors, except gender. Thus, males’ and females’ pre‐ and post‐tests scores were compared.

### 3.1 Knowledge of CNCDs

The results indicated that the HPC was effective in improving the knowledge of the students regarding CNCDS significantly with a large effect size. Students’ total knowledge score at the pre‐test [mean = 14.12; *SD* 2.95] increased significantly after the HPC (*mean* = 17.28, *SD* 1.19); *t* (177) = 14.4, *p* < .001, *d* = 1.08 (Table 2). There was a significant improvement in the students’ knowledge scores on DM, hypertension and cancer subscales after the HPC. Knowledge score on the cancer subscale was the lowest in the pre‐test but increased significantly in the post‐test.

**TABLE 2:** The pre‐test and post‐test scores of health promotion knowledge and attitudes regarding CNCDs (*n* = 178)

Scale/subscales	Pre‐test *M* (*SD*)	Post‐test *M* (*SD*)	Paired *t* test	*p*	*d* ^a^
Knowledge					
DM	4.83 (1.07)	5.71 (0.59)	10.22	<.001^*^	0.77
HTN	4.84 (1.22)	5.72 (0.56)	9.35	<.001^*^	0.70
Cancer	4.46 (1.47)	5.85 (0.59)	12.61	<.001^*^	0.95
Total	14.12 (2.95)	17.28 (1.19)	14.42	<.001^*^	1.08
Attitudes					
DM	11.34 (1.16)	11.55 (1.08)	2.627	.009^*^	0.20
HTN	10.56 (1.61)	11.25 (1.46)	5.041	<.001^*^	0.38
Cancer	10.25 (1.69)	11.48 (1.32)	8.949	<.001^*^	0.67
Total	32.15 (3.51)	34.28 (3.24)	8.216	<.001^*^	0.62

Note

Effect size (Cohen *d*) ranges: Knowledge (0.70–1.08), attitude: 0.20–0.67.

Abbreviations: DM, diabetes mellitus; HTN, hypertension; M, mean; *SD*, standard deviation.

^a^ Calculation based on Cohen's *d*; ^*^ *p* < .05.

Knowledge items with the lowest pre‐test scores had the most significant (*p* < .001) increase on the post‐test. For example, the knowledge score regarding BC risk factors was the lowest on pre‐test (*mean* 0.40; *SD* 0.49), but significantly (*p* < .001) increased by 0.55 points on the post‐test (*mean* 0.95; *SD* 0.22). Also, pre‐test scores on DM symptoms (*mean* 0.67; *SD* 0.47), DM complications (*mean* 0.51; *SD* 0.50), hypertension as a silent killer (*M* = 0.63; *SD* 0.48) and sun exposure risk factor (*M* = 0.67; *SD* 0.47) were low; these mean scores increased significantly (*p* < .001) in the post‐test to 0.96 (*SD* 0.21), 0.87 (*SD* 0.34), 0.99 (*SD* 0.12) and 0.97 (*SD* 0.18), respectively. Items with relatively high pre‐test scores also improved significantly on the post‐test. For example, scores on normal fasting blood glucose level (*mean* 0.95; *SD* 0.23), symptoms of hypoglycaemia (*mean* 0.94; *SD* 0.23) and BC (*M* = 0.92; *SD* 0.28) increased significantly (*p* = . 034, *p* = .006 and *p* < .001, respectively) to 0.99 out of 1 in the post‐test (Table 3).

**TABLE 3:** Pre‐ and post‐test scores on health promotion knowledge items (*n* = 178)

Item description	Pre‐test *M* (*SD*)	Post‐test *M* (*SD*)	*p* ^a^	Pre‐test		*p* ^b^	Post‐test		*p* ^b^
	*n* = 178	*n* = 178		Males *n* = 55	Female *n* = 123		Males *n* = 55	Female *n* = 123	
DM									
Major cause of DM	0.82 (0.38)	0.94 (0.24)	.001*	0.76 (0.43)	0.85 (0.36)	.221	0.91 (0.29)	0.95 (0.22)	.338
Symptom of DM	0.67 (0.47)	0.96 (0.21)	<.001*	0.58 (0.50)	0.72 (0.45)	.092	0.96 (0.19)	0.95 (0.22)	.714
Normal FBG level	0.95 (0.22)	0.99 (0.11)	.034*	0.91 (0.29)	0.97 (0.18)	.172	1.00 (0.00)	0.98 (0.13)	.344
Complications of DM	0.51 (0.50)	0.87 (0.34)	<.001*	0.49 (0.51)	0.51 (0.50)	.794	0.84 (0.37)	0.88 (0.33)	.455
DM risk factors	0.93 (0.25)	0.97 (0.18)	.134	0.93 (0.26)	0.93 (0.25)	.851	1.00 (0.00)	0.95 (0.22)	.014*
Hypoglycaemia symptoms	0.94 (0.23)	0.99 (0.08)	.006*	0.93 (0.26)	0.95 (0.22)	.524	1.00 (0.00)	0.99 (0.09)	.505
HTN									
HTN is the “silent killer”	0.63 (0.48)	0.99 (0.12)	<.001*	0.58 (0.50)	0.66 (0.48)	.329	0.98 (0.13)	0.98 (0.13)	.344
High BP reading	0.72 (0.45)	0.96 (0.20)	<.001*	0.76 (0.43)	0.70 (0.46)	.380	0.96 (0.19)	0.94 (0.20)	.893
HTN complications	0.87 (0.34)	0.99 (0.11)	<.001*	0.76 (0.43)	0.91 (0.29)	.023*	0.98 (0.14)	0.99 (0.09)	.559
HTN risk factors	0.95 (0.22)	0.98 (0.15)	.166	0.93 (0.26)	0.96 (0.20)	.370	0.98 (0.14)	0.98 (0.16)	.798
HTN symptoms	0.88 (0.32)	0.96 (0.22)	.015*	0.84 (0.37)	0.90 (0.30)	.250	0.96 (0.19)	0.95 (0.22)	.714
Lifelong treatment of HTN	0.79 (0.41)	0.85 (0.36)	.109	0.73 (0.45)	0.81 (0.39)	.225	0.82 (0.39)	0.86 (0.35)	.456
Cancer									
RF of BC	0.40 (0.49)	0.95 (0.22)	<.001*	0.27 (0.45)	0.46 (0.50)	.013*	0.85 (0.36)	0.99 (0.09)	.007*
Screening of BC	0.87 (0.34)	0.98 (0.13)	<.001*	0.76 (0.43)	0.92 (0.27)	.016*	0.96 (0.19)	0.99 (0.09)	.295
Cancer RFs	0.83 (0.38)	0.97 (0.17)	<.001*	0.71 (0.46)	0.89 (0.32)	.011*	0.95 (0.23)	0.98 (0.13)	.249
BC symptoms	0.92 (0.28)	0.99 (0.08)	<.001*	0.84 (0.37)	0.95 (0.22)	.037*	0.98 (0.14)	1.00 (0.00)	.322
Colon cancer symptoms	0.76 (0.43)	0.98 (0.13)	<.001*	0.67 (0.48)	0.80 (0.40)	.075	0.96 (0.19)	0.99 (0.09)	.295
Risk of Sun exposure	0.67 (0.47)	0.97 (0.18)	<.001*	0.56 (0.50)	0.72 (0.45)	.057	0.98 (0.14)	0.96 (0.20)	.446

Abbreviations: BC, breast cancer; BP, blood pressure; DM, diabetes mellitus; FBG, fast blood glucose; HTN, hypertension; M, mean; RFs, risk factors; *SD*, standard deviation.

^a^ Paired sample *t* test.

^b^ Independent sample *t* test.

* *p* < .05.

The overall and subscale knowledge scores increased significantly (*p* < .001) in the post‐test for both males and females with higher increases among males (Table 4). The overall effect size was large for males (*d* = 1.10) and females (*d* = 1.15). Comparing the scores between genders, females’ knowledge scores were higher at both tests, except for the post‐test knowledge of DM which was equal among both genders. However, pre‐test gender differences were only significant in the total score of knowledge (*p* = .004), particularly regarding cancer (*p* = .001).

**TABLE 4:** Pre‐ and post‐test scores on the knowledge and attitudes scales among males and females (*n* = 178)

Scale/subscale	Pre‐test *M* (*SD*)			Post‐test *M* (*SD*)			Pre/Post *t* test Male		Pre/Post *t* test Female	
	Male *n* = 55	Female *n* = 123	*p* ^a^	Male *n* = 55	Female *n* = 123	*p* ^a^	*t* ^b^	*p*	*t* ^b^	*p*
Knowledge										
DM	4.60 (1.27)	4.93 (0.96)	.092	5.71 (0.53)	5.71 (0.61)	.985	6.19	<.001*	8.23	<.001*
HTN	4.60 (1.49)	4.94 (1.07)	.127	5.71 (0.53)	5.72 (0.58)	.874	5.58	<.001*	7.58	<.001*
Cancer	3.82 (1.69)	4.74 (1.27)	.001*	5.69 (0.90)	5.92 (0.53)	.074	7.65	<.001*	10.5	<.001*
Total	13.02 (3.69)	14.61 (2.41)	.004*	17.12 (1.38)	17.35 (1.09)	.215	8.15	<.001*	12.8	<.001*
Attitudes										
DM	11.07 (1.39)	11.46 (1.03)	.070	11.23 (1.44)	11.68 (0.84)	.036*	1.10	.275	2.43	.017*
HTN	10.38 (1.64)	10.63 (1.60)	.336	10.98 (2.09)	11.37 (1.06)	.200	1.96	.055	5.06	<.001*
Cancer	9.69 (1.78)	10.50 (1.59)	.003*	11.11 (1.64)	11.65 (1.12)	.029*	4.78	<.001*	7.71	<.001*
Total	31.14 (3.59)	32.59 (3.41)	.011*	33.33 (4.45)	34.70 (2.42)	.035*	3.96	<.001*	7.40	<.001*

Abbreviations: DM, diabetes mellitus; HTN, hypertension; M, mean; *SD*, standard deviation.

^a^ Independent sample *t* test.

^b^ Paired sample *t* test.

* *p* < .05.

### 3.2 Attitudes of students towards CNCDs

There was a significant improvement in the overall attitudes score towards CNCDs after the course with a medium effect size. There was a significant difference in students’ total attitudes score before (*mean* 32.15, *SD* 3.51) and after the HPC (*mean* 34.28, *SD* 3.24); *t* (177) = 8.22, *p* < .001, *d* = 0.62). Scores on the cancer subscale were the lowest in the pre‐test but had the highest increase on the post‐test. Attitudes scores on each subscale are given in Table 2.

For all students, items with the lowest pre‐test scores had the most significant increase in the post‐test. The item on the chronicity of hypertension had the lowest pre‐test score *(mean* 1.40, *SD* 0.76) and showed a significant increase on the post‐test (*p* = .001). Also, the pre‐test scores on items regarding heredity of hypertension and BC and the importance of a healthy diet and regular exercise for cancer prevention significantly improved on the post‐test (*p* < .001). There was a significant improvement in students’ scores regarding the chronicity of DM (*p* = .015) and the benefit of avoiding salty and fatty diets (*p* = .032) (Table 5).

**TABLE 5:** Pre‐ and post‐test scores on attitudes items (*n* = 178)

Item description	Pre‐test *M* (*SD*)	Post‐test *M* (*SD*)	*p* ^a^	Pre‐test		*p* ^b^	Post‐test		*p* ^b^
				Male	Female		Male	Female	
DM	*n* = 178	*n* = 178		*n* = 55	*n* = 123		*n* = 55	*n* = 123	
DM is chronic non‐curative	1.73 (0.59)	1.85 (0.50)	.015*	1.56 (0.69)	1.80 (0.52)	.023*	1.73 (0.65)	1.90 (0.41)	.070
Following healthy diet, low sugar and fats	1.86 (0.45)	1.92 (0.34)	.101	1.78 (0.57)	1.89 (0.38)	.184	1.93 (0.33)	1.92 (0.35)	.878
Testing BGL	1.93 (0.31)	1.94 (0.32)	.836	1.96 (0.19)	1.92 (0.35)	.376	1.89 (0.42)	1.96 (0.27)	.266
Regular exercise and IBW	1.98 (0.18)	1.95 (0.31)	.198	1.98 (0.14)	1.98 (0.20)	.835	1.91 (0.40)	1.97 (0.25)	.320
Regular intake of DM medications	1.92 (0.37)	1.97 (0.24)	.060	1.84 (0.50)	1.95 (0.28)	.116	1.93 (0.33)	1.98 (0.18)	.231
DM is heritable	1.92 (0.29)	1.92 (0.36)	1.00	1.95 (0.23)	1.91 ( (0.31)	.460	1.85 (0.49)	1.95 (0.28)	.175
HTN									
HTN is chronic and non‐curable	1.40 (0.76)	1.66 (0.71)	.001*	1.33 (0.72)	1.44 (0.78)	.368	1.55 (0.81	1.72 (0.66)	.176
Avoid salt and fat diet	1.86 (0.47)	1.94 (0.31)	.032*	1.80 (0.59)	1.89 (0.41)	.329	1.89 (0.42)	1.97 (0.25)	.210
Regular monitor of BP	1.96 (0.23)	1.97 (0.26)	.594	1.96 (0.19)	1.95 (0.25)	.744	1.93 (0.38)	1.98 (0.18)	.295
Regular exercise and ideal body weight	1.93 (0.32)	1.98 (0.21)	.083	1.98 (0.14)	1.90 (0.37)	.039*	1.96 (0.27)	1.98 (0.18)	.559
Regular intake of HTN medications	1.92 (0.31)	0.192 (0.38)	1.00	1.87 (0.39)	1.94 (0.27)	.225	1.89 (0.46)	1.93 (0.33)	.471
HTN is heritable	1.49 (0.76)	1.78 (0.56)	<.001*	1.44 (0.71)	1.51 (0.78)	.540	1.76 (0.58)	1.78 (0.58)	.858
Cancer									
Cancer/BC is curative if early detected	1.93 (0.31)	1.98 (0.21)	.088	1.85 (0.45)	1.97 (0.22)	.080	1.96 (0.27)	1.98 (0.18)	.559
Healthy diet	1.43 (0.75)	1.80 (0.57)	<.001*	1.35 (0.78)	1.46 (0.74)	.334	1.73 (0.65)	1.83 (0.52)	.309
Breast self‐examination	1.90 (0.36)	1.98 (0.21)	.009*	1.82 (0.51)	1.94 (0.27)	.092	1.96 (0.27)	1.98 (0.18)	.559
Regular exercise	1.53 (0.70)	1.87 (0.46)	<.001*	1.45 (0.69)	1.56 (0.70)	.349	1.78 (0.57)	1.90 (0.39)	.156
Psychologic support and treatments	1.97 (0.24)	1.96 (0.27)	.836	1.98 (0.14)	1.96 (0.27)	.558	1.91 (0.40)	1.98 (0.18)	.188
Cancer/BC is heritable	1.49 (0.74)	1.90 (0.38)	<.001*	1.24 (0.77)	1.61 (0.70)	.002*	1.76 (0.54)	1.97 (0.25)	.010*

Abbreviations: BC, breast cancer; BGL, blood glucose level; BP, blood pressure; DM, diabetes mellitus; HTN, hypertension; IBW, ideal body weight; M, mean; *SD*, standard deviation.

^a^ Paired sample *t* test.

^b^ Independent sample *t* test.

* *p* < .05.

Males’ and females’ overall score towards CNCDs improved significantly (*p* < .001) in the post‐test with higher increases among males. The overall effect size was medium for males (*d* = 0.53) and females (*d* = 0.67) (Table 4). Post‐test scores on attitudes towards cancer significantly improved among both genders (*p* < .001), while the scores on DM and hypertension significantly improved (*p* = .017 and *p* < .001, respectively) only among females. Examining differences between genders, females scored higher than males on the total attitudes scale and its subscales on both tests. In the pre‐test, gender differences were significant in cancer (*p* = .003) and total attitudes (*p* = .011) scores. In post‐test, females scored significantly higher than males on the total scale (*p* = .035), DM (*p* = .036) and cancer subscales (*p* = .029).

## 4 DISCUSSION

This study aimed to assess the effect of the HPC on knowledge and attitudes about CNCDs among undergraduate students in Jordan. The main finding of this study showed that the HPC was effective in improving the overall knowledge and attitudes towards CNCDs among all the students, regardless of gender.

### 4.1 Knowledge

Results of this study indicate that the HPC was significantly effective in improving the health promotion knowledge regarding DM, hypertension and cancer among all students. The interactive education about CNCDs significantly improved students’ knowledge of general facts, risk factors, prevention strategies and healthy lifestyles, treatment and complications of CNCDs. This result is supported by a previous studies that used similar interactive methods for education about CNCDs (Alkhasawneh et al., 2017; Alsaraireh & Darawad, 2019; Selvam et al., 2017). Knowledge about common CNCDs could be beneficial for young adults in fighting the epidemic of CNCDs when entering the workforce, starting a career or establishing a new family.

Students in this study demonstrated the highest improvement in their knowledge about cancer. Their knowledge about cancer was the lowest before the HPC, particularly regarding the risk factors and warning symptoms. University students often lack pertinent knowledge regarding different types of cancer (Alsaraireh & Darawad, 2018; Asgarlou et al., 2016; Kan’an, 2018; Suleiman, 2014; Ugurlu et al., 2016). Students in this study had the lowest knowledge score on BC risk factors. Similarly, two recent Jordanian studies revealed poor knowledge of university students regarding symptoms and risk factors of BC (Alsaraireh & Darawad, 2018; Kan’an, 2018). Knowledge scores in this study are expected among students who are young and healthy (only 5.1% of students had an existing long‐term illness). Older students who have previous experience with cancer report better knowledge about cancer (Mhaidat et al., 2018). Educating young adults about the risk factors and warning signs is important for early detection and prevention of disease occurrence later in life (Joh et al., 2017).

Students’ knowledge regarding risk factors, symptoms and screenings of different types of cancer improved significantly after the HPC, which is consistent with previous findings (Abuadas & Abuadas, 2020; Alkhasawneh et al., 2017; Ouyang & Hu, 2014). Similarly, a Jordanian study found that the BC educational programme, using various teaching methods, was effective in improving university students’ knowledge regarding BC, its warning signs and risk factors (Alsaraireh & Darawad, 2019). It was important to educate students on the risk factors and prevention of cancer, particularly BC, because it was the most common type (37.3%) of cancer among Jordanian females in 2012 and the most leading cause of cancer‐related deaths among females (24.53%) (MOH, 2012).

Also, students’ knowledge of DM and hypertension improved significantly after the HPC which is similar to other reports (Nayak et al., 2016; Selvam et al., 2017; Shah et al., 2016). Emphasis on the specific topics, the use of audiovisual aids and case studies, providing explicit information on normal values, risk factors and ways to manage complications in the HPC significantly improved students’ knowledge. Students’ baseline knowledge of diabetes and hypertension symptoms, complications and general facts was low, but improved significantly; this is comparable to previous findings (Nayak et al., 2016; Selvam et al., 2017). Similarly, Jordanian college student's knowledge of the definition, risk factors, complications (e.g. kidney, eye, nervous system and lower extremities) and prevention of DM was poor (Latifeh & Khalidisy, 2012). This highlights the need for educational programmes and awareness campaigns, lectures or brochures to address this knowledge gap (Mhaidat et al., 2018). It is essential to equip young adults with useful information for CNCDs prevention, early detection and related burden reduction to positively affect their community (Latifeh & Khalidisy, 2012).

Both male and female students’ overall knowledge regarding CNCDs significantly increased. The HPC was effective in improving their knowledge regarding DM, hypertension and cancer. The overall baseline knowledge regarding CNCDs, particularly cancer was significantly lower among males; nonetheless, this difference disappeared in the post‐test. Similar findings were reported in an Indian study where baseline knowledge regarding CNCDs was lower among males, but the improvement was significant among both genders after short training (Selvam et al., 2017). This gender difference could be explained from societal and cultural perspectives that Jordanian women generally hold more caring roles for ill family members (Shaheen et al., 2015) and may seek more information regarding diseases and treatment modalities. Jordanian females were reported to be more aware of hypertension and reproductive health (Ali et al., 2018; Jaddou et al., 2011). This gender difference regarding CNCD knowledge should be taken into consideration when providing health education to young adults.

### 4.2 Attitudes

The study showed a significant improvement in students’ overall attitudes regarding CNCDs and related health‐promoting lifestyles after the HPC, which is consistent with previous findings (Alsaraireh & Darawad, 2019; Ouyang & Hu, 2014; Selvam et al., 2017). Such improvement demonstrates another success for the HPC. Improving attitudes at an early stage is very significant, as positive attitudes were associated with health‐promoting practices that reduce disease occurrence (Dayal & Singh, 2018).

In general, students in this study demonstrated the highest improvement in their attitudes towards cancer, followed by hypertension, then DM. This might be explained by students' baseline knowledge and attitudes, where cancer scores were the lowest. A positive association between knowledge and attitudes scores was reported in previous studies (Dayal & Singh, 2018; Shen et al., 2017). The HPC was effective in improving students’ attitudes regarding risk factors and the preventive and screening measures of cancer, such as the heredity risk of cancer, the importance of healthy diets and regular exercise in cancer prevention and the importance of breast self‐examination for early detection of BC. Similar positive impact of an educational programme on Jordanian students’ attitudes towards BC was reported by Alsaraireh and Darawad (2019). University students are vulnerable to engage in risky behaviours and; therefore, health education is a priority to improve their attitudes towards health‐promoting behaviours and reduce their risk for developing diseases later in life (Dayal & Singh, 2018; Joh et al., 2017).

The HPC was effective in improving students’ attitudes regarding different aspects of diabetes and hypertension, such as their chronic and hereditary nature. This could be explained by the improvement in students’ knowledge of the aetiology of these diseases, as a positive association between knowledge and attitudes towards CVDs among young adults has been reported (Dayal & Singh, 2018). Students’ attitudes towards the importance of low‐salt and low‐fat diets intake to prevent hypertension also improved after the course which emphasized on healthy nutrition and consequences of poor dietary habits. Similar improvement in attitudes regarding diabetes and hypertension by educational interventions was reported (Eunice, 2017; Selvam et al., 2017). Appropriate and planned strategies are required to improve students’ knowledge and attitudes about CNCDs since students are at a critical developmental phase of taking responsibility for their personal health and establishing lifelong healthy behaviours (Joh et al., 2017).

Both male and female students’ overall attitudes regarding CNCDs significantly improved after the HPC, particularly towards cancer among males and the three diseases among females. Such improvement was reported by others (Alsaraireh & Darawad, 2019; Eunice, 2017; Ouyang & Hu, 2014; Selvam et al., 2017). In general, female students demonstrated better attitudes regarding CNCDs, DM, hypertension and cancer, which is consistent with previous studies (Fatema et al., 2017; Shen et al., 2017). From a societal and cultural perspective, Jordanian females generally care for ill family members (Shaheen et al., 2015) thus developing a more positive attitude towards these diseases. Jordanian males’ attitudes towards BC could be attributed to their perception that BC is rare in men. Improving males’ attitudes towards BC is important in encouraging female relatives seeking BC screening (Al Dasoqi et al., 2017) because negative attitudes towards BC were reported among young females (Alsaraireh & Darawad, 2019). Health education regarding cancer and other CNCDs is crucial for young adults (Al Dasoqi et al., 2017; Parmar et al., 2014) and including these topics in the curriculum may help in early detection and disease prevention (Abdo et al., 2019; Alsaraireh & Darawad, 2018). Furthermore, designing gender‐specific strategies for future health campaigns can address issues related to gender differences (Shen et al., 2017).

### 4.3 Limitations

This study has some limitations associated with using a convenience sample and one geographical location; therefore, caution must be taken in generalizing our findings beyond the sample or to other settings. Also, using a self‐report tool may introduce recall bias, limiting the generalizability of our findings. As for strengths, this study provided information on the baseline knowledge and attitudes of students in Jordan, which can be used to design interventional programmes and innovative courses to fill the gap in knowledge and enhance the attitudes towards CNCDs. Future studies that fully assess the health promotion practices of university students are recommended. Future studies could compare students’ learning outcomes concerning specific teaching methodologies.

## 5 CONCLUSION

The findings demonstrated the effectiveness of the HPC in improving university students’ knowledge and attitudes regarding CNCDs. It is important to integrate health education regarding CNCDs and other health problems into university curricula using innovative approaches to enhance healthy behaviours in young adults. Raising awareness about specific health problems among university students can increase their responsibility towards their own health by adopting healthy lifestyles to reduce their risk for developing diseases later in life. Interactive health education provides students with essential knowledge and strategies to control CNCDs and emphasizes the value of making healthy decisions to achieve a healthier lifestyle. This can empower students in promoting their health, influencing their families’ health and consequently improving the quality of life of the emerging Jordanian workforce and community.

## CONFLICT OF INTERESTS

The authors declare that they have no competing interests.

## AUTHORS’ CONTRIBUTIONS

MA and RA contributed to the concept and design of the study. MA, MR, FA, KA, AA and RA contributed to the implementation of the intervention. MA, RA and AA were responsible for data collection. MA was responsible for data analysis and interpretation of the results, and MA and MR contributed to critically revising the methods and statistics section of the manuscript. MA, MR, FA, KA, AA and RA contributed to drafting and/or critically revising the manuscript. All authors read and approved the final version of the manuscript.

## Data Availability

This manuscript does not include any supporting information files for publication.
